# Protective Effect of Arzanol against H_2_O_2_-Induced Oxidative Stress Damage in Differentiated and Undifferentiated SH-SY5Y Cells

**DOI:** 10.3390/ijms25137386

**Published:** 2024-07-05

**Authors:** Franca Piras, Valeria Sogos, Federica Pollastro, Antonella Rosa

**Affiliations:** 1 Department of Biomedical Sciences, University of Cagliari, 09042 Monserrato, Italy; fpiras@unica.it; 2 Department of Pharmaceutical Sciences, University of Eastern Piedmont “Amedeo Avogadro”, 28100 Novara, Italy; federica.pollastro@uniupo.it

**Keywords:** neurodegenerative diseases, oxidative stress, arzanol, SH-SY5Y neuroblastoma cells, hydrogen peroxide, apoptosis

## Abstract

Oxidative stress can damage neuronal cells, greatly contributing to neurodegenerative diseases (NDs). In this study, the protective activity of arzanol, a natural prenylated α-pyrone-phloroglucinol heterodimer, was evaluated against the H_2_O_2_-induced oxidative damage in trans-retinoic acid-differentiated (neuron-like) human SH-SY5Y cells, widely used as a neuronal cell model of neurological disorders. The pre-incubation (for 2 and 24 h) with arzanol (5, 10, and 25 μM) significantly preserved differentiated SH-SY5Y cells from cytotoxicity (MTT assay) and morphological changes induced by 0.25 and 0.5 mM H_2_O_2_. Arzanol reduced the generation of reactive oxygen species (ROS) induced by 2 h oxidation with H_2_O_2_ 0.5 mM, established by 2′,7′-dichlorodihydrofluorescein diacetate assay. The 2 h incubation of differentiated SH-SY5Y cells with H_2_O_2_ determined a significant increase in the number of apoptotic cells versus control cells, evaluated by propidium iodide fluorescence assay (red fluorescence) and NucView^®^ 488 assay (green fluorescence). Arzanol pre-treatment (2 h) exerted a noteworthy significant protective effect against apoptosis. In addition, arzanol was tested, for comparison, in undifferentiated SH-SY5Y cells for cytotoxicity and its ability to protect against H_2_O_2_-induced oxidative stress. Furthermore, the PubChem database and freely accessible web tools SwissADME and pkCSM-pharmacokinetics were used to assess the physicochemical and pharmacokinetic properties of arzanol. Our results qualify arzanol as an antioxidant agent with potential neuroprotective effects against neuronal oxidative stress implicated in NDs.

## 1. Introduction

Neurodegenerative diseases (NDs) are age-related disorders that result in the death of specific types of neural cells [1]. NDs become more frequent as the average age of the population increases, presenting a serious global health problem [2]. Oxidative stress contributes to the damage and death of neuronal cells in NDs, including amyotrophic lateral sclerosis (ALS), Parkinson’s disease (PD), and Alzheimer’s disease (AD) [1,3,4].

Oxidative stress, in a biological system, is an imbalance between oxidants and antioxidants due to either high levels of reactive oxygen species (ROS) and nitrogen species (RNS), or defective function of the antioxidant system [5,6,7]. Oxidative eustress is a physiological and essential, not harmful, part of redox control/signaling, while oxidative distress represents a supraphysiological deviation [7,8]. The exposure to high supraphysiological oxidant (ROS, RNS) levels addresses unspecific targets and can damage all biomacromolecules, leading to the oxidation of nucleic acids, protein misfolding/aggregation, lipid peroxidation, disruption of integrity/cell function, and cell apoptosis induction [1,3,7,9].

Excessive ROS production has been established as the central factor of redox-regulated events disrupted in NDs [10]. The brain consumes a significant oxygen amount and is highly rich in lipids (cholesterol, sphingolipids, and glycerophospholipids). Neuronal membranes are susceptible to free radical attack and oxidative stress due to their high levels of polyunsaturated fatty acids [5,11,12]. Lipid peroxidation markers (malondialdehyde and 4-hydroxynonenal) have been detected in the substantia nigra of PD patients and the cortex and hippocampus of AD patients [12]. Moreover, an elevated level of protein nitration has been evidenced in the neocortex and hippocampus of patients with AD [12]. In addition, excess oxidative stress leads to aggregation and accumulation of specific proteins (α-synuclein protein of Lewy bodies, amyloid β-peptides, and amyloid precursor protein), which activate transcription factors in microglia and astrocytes. Sequentially, the induction of ROS, NAPDH oxidase, iNOS, COX-2, proinflammatory cytokines, and inflammatory mediators damage neurons, causing neurodegenerative disease [13]. Modifications of antioxidant/oxidant homeostasis due to excessive ROS generation, mitochondrial dysfunction, elevation of iron and nitric oxide, and activation of apoptotic pathways result in neurodegeneration in brain cells [14].

The ND prevention and therapy may be greatly aided by natural antioxidants [15]. Studies have shown that antioxidant phytochemicals in fruits and vegetables could prevent NDs in animal and cell culture models [13]. Plant-derived antioxidant compounds are considered potential therapeutic candidates for AD [16,17]. A positive relationship has been evidenced between the consumption of curcumin, resveratrol, and green tea catechins and AD prevention [13]. Resveratrol and flavonoids appear to be dietary components with specific neuroprotective effects and positive impacts on human cognitive decline due to their antioxidant properties [16,18,19].

**Figure 1:**
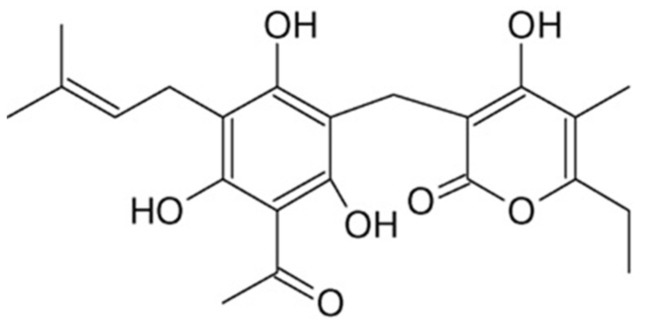
Chemical structure of arzanol.

Among phytochemicals, arzanol (Figure 1) is a naturally prenylated α-pyrone–phloroglucinol heterodimer isolated from the aerial parts of the *Helichrysum italicum* ssp. *microphyllum* (synonym of *H. microphyllum* Cambess. subsp *tyrrhenicum* Bacch., Brullo and Giusso) [20], endemism of Sardinia, Corsica, and Balearic Island. This phenol has noteworthy anti-inflammatory, anticancer, antimicrobial, antiviral, and antioxidant effects [21,22,23,24,25,26,27,28,29,30].

Arzanol contains three phenolic -OH and a prenyl group in the aromatic ring, and an enolic hydroxyl in the α-pyrone ring [21,26] (Figure 1). This phloroglucinol demonstrated radical scavenging activity in various in vitro lipid peroxidation systems [26,27,29], and significant antioxidant effects in cell and animal models of oxidative stress [27,29,30]. Recently, its ability to counteract cytotoxicity, ROS generation, mitochondrial membrane depolarization, and apoptosis has been demonstrated in a model of oxidative damage, induced by hydrogen peroxide (H_2_O_2_), in keratinocytes [30].

Arzanol has been identified (by MS-based chemical proteomics) as a positive modulator of brain glycogen phosphorylase (bGP), the enzyme that promotes brain glycogen mobilization, qualifying this phenol as a potential therapy to combat neurodegenerative conditions [24]. Moreover, arzanol demonstrated, in glutamate-exposed SH-SY5Y cells, an inhibitory effect on the activity of silent information regulator 1 (SIRT1), an enzyme linked with neuroinflammation in neurons and glial cells [31].

The current study aimed to evaluate, for the first time, the antioxidant potential of arzanol against H_2_O_2_-induced oxidative damage in differentiated (neuron-like) SH-SY5Y cells, a cell model amply used to study neuronal oxidative stress and the protective effects of antioxidant compounds [14,32]. H_2_O_2_ is a small electroneutral molecule [33] and represents the major redox-signaling molecule in cells [7,8,10]. This molecule, at supraphysiological concentrations, is widely employed to induce a condition of oxidative “distress”, causing oxidative damage in cellular models, including neuronal cells [7,8,14,32,34,35].

The SH-SY5Y human neuroblastoma cell line is extensively used in neuroscience research [36]. Undifferentiated (neuroblast-like cells) and differentiated (neuron-like) SH-SY5Y cells are largely used as in vitro neuronal cell models of neurological disorders [37,38]. Undifferentiated SH-SY5Y cells are characterized by polygonal cell shape and short processes. They express markers of immature neuronal cells, transcriptional regulators [39], and low levels of dopaminergic markers, including dopamine transporter, dopamine-β-hydroxylase, and tyrosine-hydroxylase (TH) [37,38]. However, the morphology and functionality of undifferentiated SH-SY5Y cells differ from mature neurons. SH-SY5Y cells are induced to differentiate in a more neuron-like phenotype by trans-retinoic acid (RA). The differentiated cells are characterized by a fusiform shape, neurite outgrowth, increased levels of TH, and neuronal markers (synaptophysin, synaptic-associated proteins, and neuron-specific enolase), and they mimic neuronal response properties in experimental models [40,41].

In this study, the experimental design was performed to verify the arzanol ability to protect RA-differentiated neuronal SH-SY5Y cells against cytotoxicity, morphological changes, and ROS generation induced by supraphysiological concentrations of H_2_O_2_. The protective effect of the arzanol pre-treatment against H_2_O_2_-induced apoptosis, observed in differentiated SH-SY5Y cells as the consequence of peroxide oxidation, was also monitored. In addition, arzanol was tested, for comparison, in undifferentiated SH-SY5Y cells for cytotoxicity and its ability to protect against H_2_O_2_-induced oxidative damage. The results of this study were expected to provide useful indications on the arzanol potential as a therapeutic intervention against neuronal oxidative stress implicated in NDs.

## 2. Results

### 2.1. Cell Differentiation

**Figure 2:**
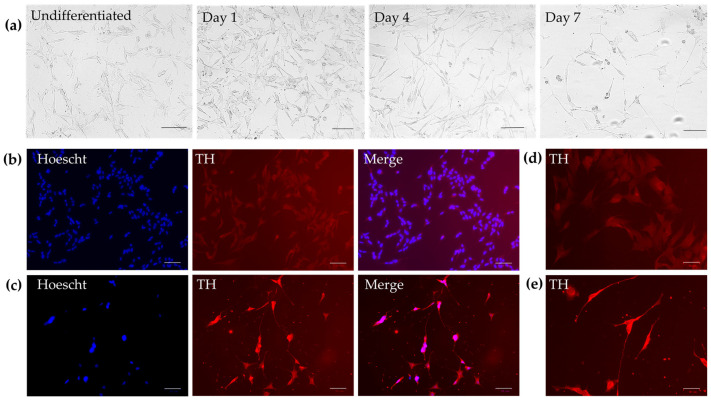
Phase contrast microscopy images (scale bar = 100 µm) of SH-SY5Y undifferentiated cells at seeding and during 7 days of treatment with 10 μM trans-retinoic acid (the process of differentiation) (**a**). Nuclear staining with Hoechst (blue), immunofluorescent staining of tyrosine hydroxylase (TH, red), and merge-colocalization (bar = 78 µm) in undifferentiated (**b**) and 7-days differentiated (**c**) SH-SY5Y cells. Magnifications (bar = 39 µm) of TH staining in undifferentiated (**d**) and 7-days differentiated (**e**) SH-SY5Y cells.

The morphology and protein expression differences of undifferentiated and differentiated SH-SY5Y cells are reported in Figure 2.

Figure 2a shows phase contrast microscopy images of SH-SY5Y undifferentiated cells at seeding and during 7 days of the differentiation process induced by 10 μM RA treatment. Undifferentiated cells quickly grew, occasionally in aggregates, and showed a non-polarized shape with short processes. The differentiated SH-SY5Y cells, during the differentiation process, showed a reduced proliferation rate and grew long, mostly exhibiting a fusiform shape, with neurite projections occasionally branched.

Figure 2b,c show nuclear staining with Hoechst (blue), immuno-fluorescent staining of TH (a marker of dopaminergic neurons, red), and merge-colocalization in undifferentiated and 7-day differentiated SH-SY5Y cells, respectively. Undifferentiated neuroblastoma cells expressed a weak TH staining intensity (Figure 2b,d), while differentiated SH-SY5Y cells (Figure 2c,e) exhibited a marked red fluorescence, indicating a process of neuronal dopaminergic differentiation.

### 2.2. Effect of H_2_O_2_ on SH-SY5Y Cell Viability (MTT Assay)

**Figure 3:**
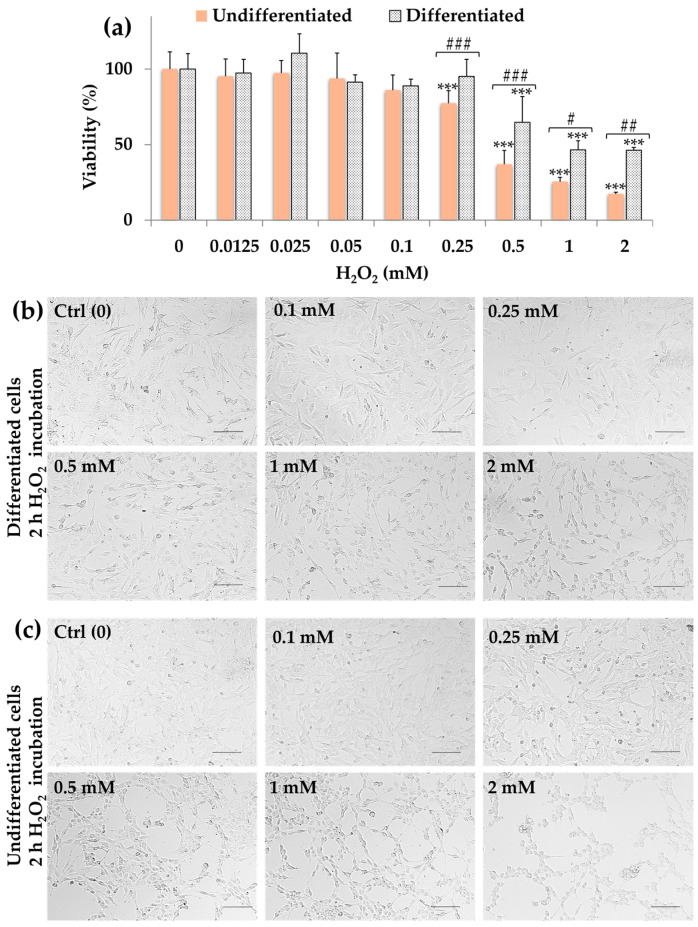
(**a**) Viability, expressed as % of the control (0), induced by incubation for 24 h with different amounts of H_2_O_2_ (0.0125 to 2 mM) in differentiated and undifferentiated SH-SY5Y cells (MTT assay). Data are presented as mean and standard deviation (SD) of three independent experiments involving quadruplicate analyses for each sample (n = 12). The statistical significance of differences was assessed by two-way ANOVA followed by the Tukey multiple comparisons test. For each series (undifferentiated and differentiated SH-SY5Y cells), *** = *p* < 0.001 versus the respective Ctrl. For each H_2_O_2_ concentration, ^###^ = *p* < 0.001, ^##^ = *p* < 0.01, and ^#^ = *p* < 0.05 for differentiated versus undifferentiated SH-SY5Y cells. The panels show representative images of phase contrast of control (Ctrl) and SH-SY5Y cells 24 h treated with the different amounts of H_2_O_2_ (0.1–2 mM) measured in differentiated (**b**) and undifferentiated (**c**). Bar = 100 μm.

The colorimetric MTT assay, a method used for evaluating cell viability, proliferation, and cytotoxicity, was employed to assess the H_2_O_2_ impact on differentiated SH-SY5Y cell viability [29,30]. The H_2_O_2_ cytotoxicity was also evaluated in undifferentiated SH-SY5Y cells for comparison. Cell viability data (expressed as a percentage of the control), by MTT assay, measured after 24 h incubation with various H_2_O_2_ concentrations (from 0.0125 to 2 mM) in differentiated and undifferentiated SH-SY5Y cells are shown in Figure 3a.

Figure 3b,c display the corresponding morphological images of differentiated and undifferentiated SH-SY5Y cells, respectively, observed under phase contrast microscopy.

Cell viability values (Figure 3a) remained constant in H_2_O_2_-exposed differentiated neuronal cells, compared to the control cells, when the oxidant concentration was between 0.0125 and 0.25 mM. However, a significant concentration-dependent cytotoxic effect (*p* < 0.001 versus control cells) was observed at H_2_O_2_ concentrations from 0.5 to 2 mM, resulting in a 35–54% reduction in cell viability.

Microscopic observations (Figure 3b) evidenced no significant changes in cell morphology or density in differentiated SH-SY5Y cells within the H_2_O_2_ dose range from 0.0125 to 0.25 mM. However, at higher H_2_O_2_ concentrations, decreased cell density areas were observed, and the cells showed a reduction in their cellular size, particularly at 1 and 2 mM.

The exposure of undifferentiated neuroblastoma cells to H_2_O_2_ (Figure 3a) did not significantly change the cell viability percentage, compared to the control cells, when the oxidant concentration was between 0.0125 and 0.1 mM. However, H_2_O_2_ caused a significant concentration-dependent cytotoxic effect in undifferentiated cells starting from 0.25 mM, leading to a reduction (ranging from 23% to 83%) in cell viability.

Microscopic observations (Figure 3c) did not reveal any significant alteration in neuroblastoma cell morphology or density within the H_2_O_2_ dose range of 0.0125–0.05 mM. However, at higher concentrations, areas with decreased cell density were observed, and the cells displayed changes in size, with a reduced cytoplasm volume, especially at the highest H_2_O_2_ concentrations.

Interestingly, H_2_O_2_ emerged as significantly more cytotoxic in undifferentiated than in differentiated SH-SY5Y cells from the dose of 0.25 mM.

### 2.3. Effect of Arzanol on SH-SY5Y Cell Viability (MTT Assay)

**Figure 4:**
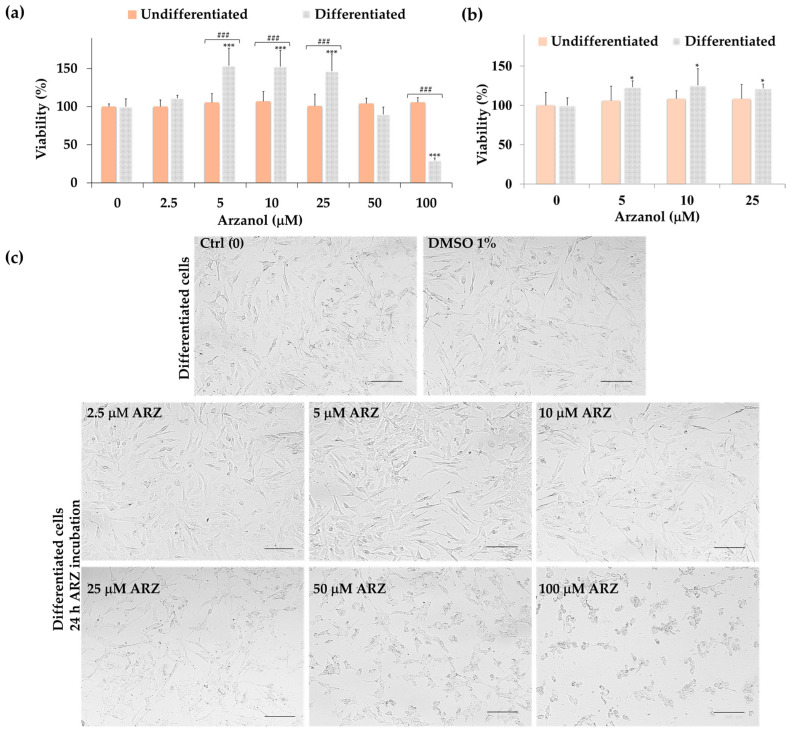
Viability, expressed as % of the control (0), induced by incubation for 24 h (**a**) and 2 h (**b**) with different amounts of arzanol (2.5–100 μM) in differentiated and undifferentiated SH-SY5Y cells (MTT assay). All data are presented as mean and standard deviation (SD) of three independent experiments involving six replicates for each sample (n = 18). The statistical significance of differences was assessed by two-way ANOVA followed by the Tukey multiple comparisons test. For each series (undifferentiated and differentiated SH-SY5Y cells), *** = *p* < 0.001 and * = *p* < 0.05 versus the respective Ctrl. For each arzanol concentration, ^###^ = *p* < 0.001 for differentiated versus undifferentiated SH-SY5Y cells. Panel (**c**) shows representative phase contrast images of differentiated SH-SY5Y control cells (Ctrl) and cells treated for 24 h with vehicle (DMSO 1%) and different amounts of arzanol. Bar = 100 μm.

Then, the cytotoxic effects of arzanol on differentiated SH-SY5Y cells, after 24 h of incubation at various concentrations (2.5–100 μM), were determined by MTT assay. The results, expressed as a percentage of the control, are shown in Figure 4a. The cytotoxicity of arzanol in undifferentiated SH-SY5Y cells is also reported for comparison in Figure 4a.

No significant difference in cell viability was observed in differentiated neuronal cells after the treatment (24 h) with 2.5 μM arzanol, in comparison to the control group. However, a significant increase in cell viability (*p* < 0.001 versus control cells) was observed in the 5–25 μM range of arzanol, reaching up to 50% higher than the control cells. Then, the viability of cells decreased with increasing concentrations of arzanol, and a viability reduction of 71%, compared to control cells, was detected at arzanol 100 μM.

Figure 4c shows the representative images observed under phase contrast microscopy of differentiated SH-SY5Y cells treated with arzanol. Moreover, images of SH-SY5Y cells treated for 24 h with vehicle (DMSO 1%, maximal tested dose), used to dissolve arzanol, are also reported. The arzanol treatment did not cause any changes in cell morphology at the lowest tested dose (2.5 μM) versus control cells, whereas the cells treated with 5–25 μM arzanol showed a greater number of fusiform and branched cells. After treatment with 50 and 100 μM arzanol, the cells showed marked changes in morphology, reduced size, and severe cell damage.

It is important to note that DMSO was not toxic in differentiated SH-SY5Y cells after 24 h of incubation, showing a 93% cell viability at the maximal tested dose (1%), without change in cell morphology versus control cells.

Conversely, arzanol did not reduce cell viability in undifferentiated SH-SY5Y cells, compared to the control cells, at all tested concentrations (Figure 4a).

Contrast microscopy images (Figure S1) showed that the 24 h arzanol treatment did not cause any significant changes in cell morphology of undifferentiated SH-SY5Y cells, without reduction in the cell number across the range of 2.5–100 μM, and the treated undifferentiated cells were very similar to control cells.

A significant cell viability increase (*p* < 0.05 versus control cells) was also observed in differentiated SH-SY5Y cells (Figure 4b) after 2 h of incubation with arzanol at the dose range 5–25 μM. No cytotoxicity was evidenced for the phenol in neuroblastoma undifferentiated cells in the same experimental conditions.

Taking into consideration the results obtained in the viability assay, the 2 h incubation period with arzanol was chosen for further assessments.

### 2.4. Protective Role of Arzanol against H_2_O_2_ Cytotoxicity

Then, the 5, 10, and 25 μM arzanol concentrations were selected to evaluate the compound protection, by MTT assay, against the cytotoxicity induced by H_2_O_2_ in differentiated SH-SY5Y cells, versus undifferentiated ones.

**Figure 5:**
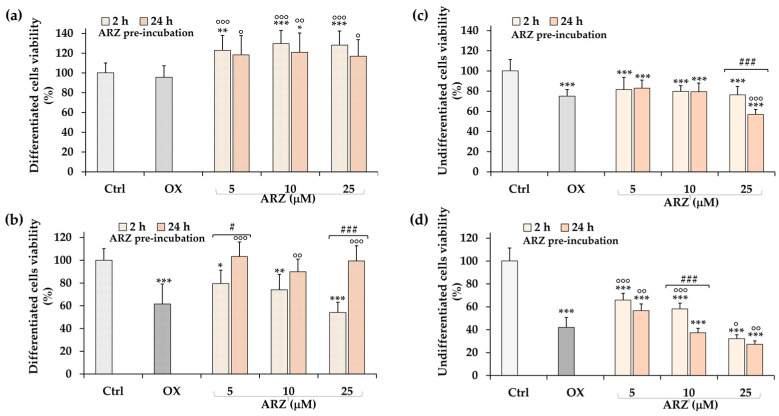
Viability (MTT assay), expressed as % of control cells (Ctrl), induced by incubation for 2 h with 0.25 mM and 0.5 mM H_2_O_2_ in differentiated (**a**,**b**, respectively) and undifferentiated (**c**,**d**, respectively) SH-SY5Y cells, measured in oxidized samples (OX) and samples oxidized after pre-incubation for 2 and 24 h with arzanol (5, 10, and 25 μM). All data are presented as mean and standard deviation (SD) of three independent experiments involving quadruplicate analyses for each sample (n = 12). The statistical significance of differences was assessed by two-way ANOVA followed by the Tukey multiple comparisons test. For each series (2 and 24 h arzanol pre-incubation), *** = *p* < 0.001, ** = *p* < 0.01, and * = *p* < 0.05 versus Ctrl; °°° = *p* < 0.001, °° = *p* < 0.01, and ° = *p* < 0.05 versus OX. For each arzanol concentration, ^###^ = *p* < 0.001 and ^#^ = *p* < 0.05 for 2 h arzanol pre-incubation versus 24 h arzanol pre-incubation.

Figure 5 displays the viability (expressed as % of control cells) measured in differentiated and undifferentiated SH-SY5Y control and cells oxidized for 2 h with H_2_O_2_ (0.25 and 0.5 mM) in the absence of arzanol (oxidized samples) and after 2 and 24 h of pre-incubation with the compound (5, 10, and 25 μM).

The treatment of differentiated SH-SY5Y cells with 0.25 mM H_2_O_2_ induced a very slight decrease in viability (Figure 5a). However, the differentiated cells pre-treated for 2 or 24 h with arzanol showed an increase in cell viability, like that observed in the absence of H_2_O_2_ (as reported in Figure 4a,b). Values of viability in the range of 123–128% were measured in differentiated cells after 2 h incubation with arzanol, while lower values were obtained after 24 h incubation.

A strong viability reduction (*p* < 0.001 versus control cells) was observed in differentiated SH-SY5Y cells after exposure to 0.5 mM H_2_O_2_ (Figure 5b). A significant protective effect against 0.5 mM H_2_O_2_-induced viability reduction was observed in differentiated neuronal cells pre-incubated for 24 h with all three arzanol concentrations. A certain, not statistically significant, protection was also observed after 2 h incubation with 5 and 10 μM arzanol.

A significant viability reduction (*p* < 0.001 versus control cells) was observed in undifferentiated SH-SY5Y cells after exposure to 0.25 mM (Figure 5c) and 0.5 mM H_2_O_2_ (Figure 5d). At the lowest H_2_O_2_ dose, arzanol did not protect cells against peroxide toxicity. The compound significantly protected undifferentiated cells from the 0.5 mM H_2_O_2_-induced cytotoxic effect at 5 μM, after 2 and 24 h of incubation, and at 10 μM after 2 h incubation.

The vehicle DMSO, at the maximal tested dose (0.25%), did not protect differentiated and undifferentiated SH-SY5Y cells (after 2 and 24 h of incubation) against the viability reduction induced by 2 h incubation with 0.25 and 0.5 mM H_2_O_2_.

### 2.5. Protective Role of Arzanol against H_2_O_2_-Induced ROS Generation

**Figure 6:**
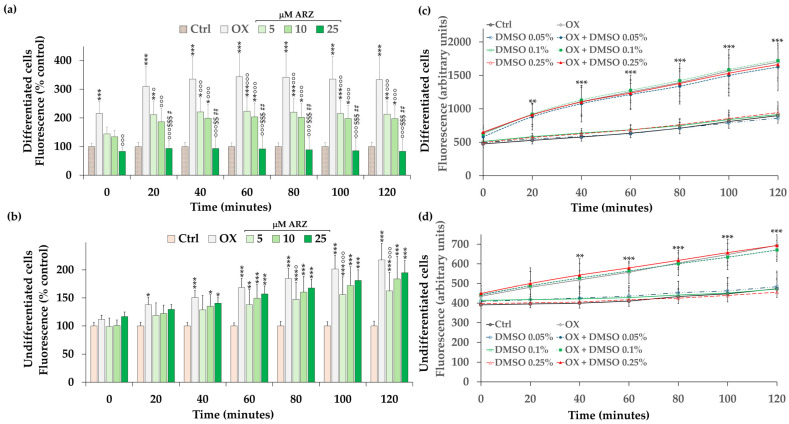
ROS-induced fluorescence measured for 2 h, at different time points, in control cells (Ctrl) and cells exposed to 0.5 mM H_2_O_2_ in the absence (oxidized samples, OX) and in the presence (after 2 h pre-incubation) of arzanol (5, 10, and 25 μM) in differentiated (**a**) and undifferentiated (**b**) SH-SY5Y cells. ROS-induced fluorescence measured for 2 h in Ctrl, cells treated for 2 h with the vehicle DMSO (0.05, 0.1, and 0.25%), and cells exposed to 0.5 mM H_2_O_2_ in the absence (OX samples) and in the presence (after 2 h pre-incubation) of DMSO (0.05, 0.1, and 0.25%) in differentiated (**c**) and undifferentiated (**d**) SH-SY5Y cells. All data are presented as mean and standard deviation (SD) of three independent experiments involving five replicates for each sample (n = 15). The statistical significance of differences was assessed by two-way ANOVA followed by the Tukey multiple comparisons test. At each time point: *** = *p* < 0.001, ** = *p* < 0.01, and * = *p* < 0.05 versus the respective control; °°° = *p* < 0.001 and ° = *p* < 0.05 versus the respective OX samples; ^$$$^ = *p* < 0.001 and ^$$^ = *p* < 0.01 versus 5 μM arzanol; ^##^ = *p* < 0.01 and ^#^ = *p* < 0.05 versus 10 μM arzanol. In panels (**c**,**d**), all types of H_2_O_2_-treated cells significantly differ from control cells.

The effect of arzanol against the ROS generation induced by the treatment with H_2_O_2_ was comparatively evaluated in differentiated and undifferentiated SH-SY5Y cells (Figure 6) using the H_2_DCFDA assay [30,42]. 

Figure 6 displays the ROS-induced fluorescence levels, expressed as % of the control, measured during 2 h in control cells, cells exposed to 0.5 mM H_2_O_2_ in the absence (oxidized samples) and in the presence (after 2 h incubation) of arzanol (5, 10, and 25 μM), in differentiated (Figure 6a) and undifferentiated (Figure 6b) SH-SY5Y cells.

The effect (after 2 h of pre-incubation) of different amounts of the vehicle DMSO (0.05, 0.1, and 0.25%) in control cells and cells treated for 2 h with 0.5 mM H_2_O_2_ is reported in Figure 6c,d for differentiated and undifferentiated SH-SY5Y cells, respectively.

In differentiated cells (Figure 6a), the treatment with H_2_O_2_ induced a significant (*p* < 0.001) noteworthy time-dependent increase in ROS generation versus control cells (3 times of control level). Pre-incubation for 2 h with arzanol significantly reduced ROS production (*p* < 0.001) in differentiated SH-SY5Y cells at all concentrations (5, 10, and 25 μM) and time points, in a dose-dependent manner.

ROS production induced by 0.5 mM H_2_O_2_ was less marked in undifferentiated cells (Figure 6b) than in differentiated ones. A significant time-dependent ROS increase (*p* < 0.001 versus control cells from 40 min) was observed after H_2_O_2_ treatment in undifferentiated cells. Cell treatment with arzanol decreased H_2_O_2_-induced ROS production in undifferentiated cells at all tested concentrations. This protective effect was inversely correlated to the compound amount and was significant, versus oxidized samples, only at 5 μM arzanol.

Pre-incubation (2 h) of differentiated (Figure 6c) and undifferentiated (Figure 6d) SH-SY5Y cells with DMSO (0.05, 0.1, and 0.25%) before H_2_O_2_-exposure (2 h) did not reduce H_2_O_2_-induced ROS production, versus oxidized control samples, at all tested concentrations.

The ROS level measured in differentiated (Figure S2a) and undifferentiated (Figure S2b) SH-SY5Y cells treated with only arzanol (5, 10, and 25 μM) was comparable to the fluorescence basal level of control cells. Fluorescence values measured in differentiated (Figure 6c) and undifferentiated (Figure 6d) SH-SY5Y cells treated (2 h of incubation) with DMSO (0.05, 0.1, and 0.25%) were identical to those measured in control cells, evidencing no vehicle protection against basal ROS generation.

### 2.6. Protective Effect of Arzanol against Apoptosis

The arzanol protective effect on cell death and apoptosis induced by H_2_O_2_ cell oxidation was assessed by staining differentiated neuronal SH-SY5Y cells with propidium iodide (PI), a DNA-binding fluorescent (red) dye that evidences necrotic and late apoptotic cells [43], and with NucView 488 (NV) dye, an enzyme caspase-3 substrate, capable of detecting the activity of caspase-3/7 within cells [40].

**Figure 7:**
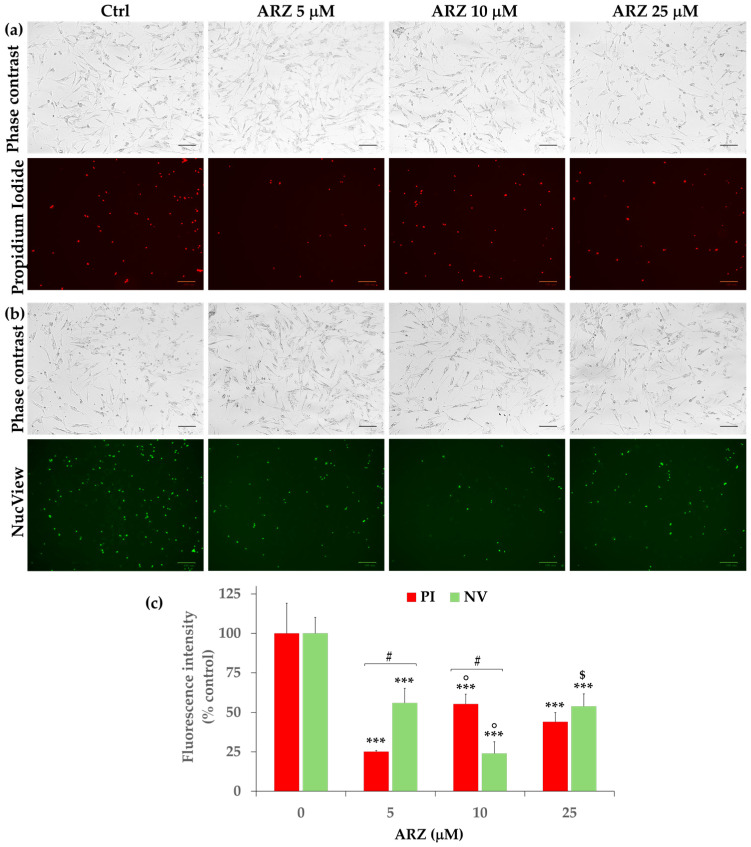
Red emission images, as revealed by propidium iodide (PI) fluorescence (**a**), green emission images (NucView 488 fluorescence, NV) (**b**), and the corresponding phase contrast images measured in differentiated SH-SY5Y control cells (0) and in cells 2 h treated with arzanol 5, 10 and 25 μM (scale bar of microscopy images = 100 μm). Intensity of red (PI) and green (NV) emission fluorescence, expressed as % control, after arzanol treatment as obtained by image analysis (**c**). All data are presented as mean and standard deviation (SD) of three independent experiments involving duplicate analyses for each sample (n = 6). The statistical significance of differences was assessed by two-way ANOVA followed by the Tukey multiple comparisons test. For each series, *** = *p* < 0.001 versus the respective control cells (0); ° = *p* < 0.05 versus 5 μM arzanol; ^$^ = *p* < 0.05 versus 10 μM arzanol. For each arzanol concentration, ^#^ = *p* < 0.05 for PI versus NV.

Figure 7 shows the phase contrast, red emission (by PI) (Figure 7a), and green emission (NV) (Figure 7b) images obtained for differentiated SH-SY5Y control cells and cells treated for 2 h with 5, 10 and 25 μM arzanol, while the quantitative data of PI and NV intensity emission fluorescence, expressed as % control, are depicted in Figure 7c.

A significant decrease (*p* < 0.001) in the red and green emission was observed in differentiated SH-SY5Y cells treated for 2 h with pure arzanol (5, 10, and 25 μM) compared to the basal level of control cells, demonstrating the phloroglucinol protection against the apoptotic process normally present in differentiated SH-SY5Y cells.

Some differences were observed between the two methods at 5 and 10 μM arzanol.

**Figure 8:**
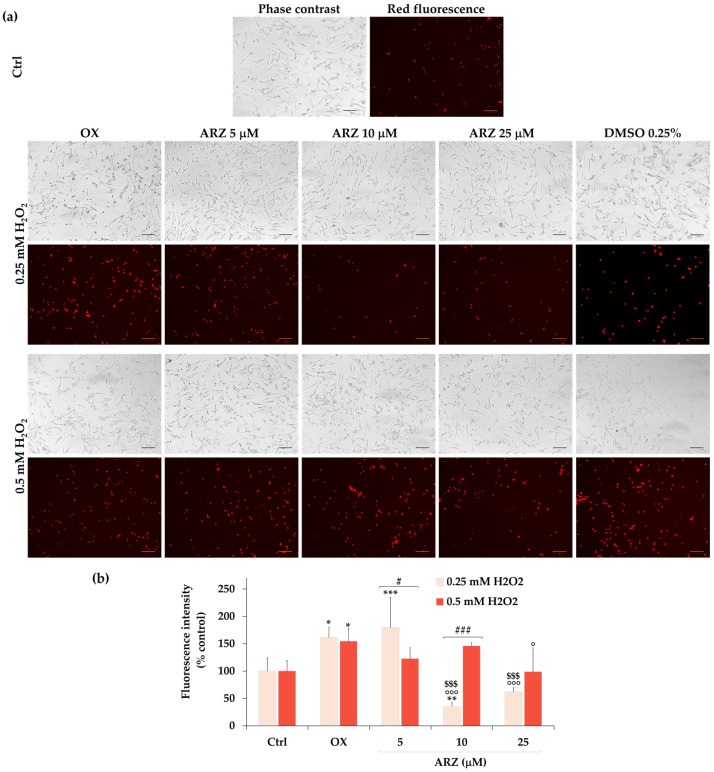
Phase contrast and fluorescence red emission images, by propidium iodide (PI) assay, obtained in differentiated SH-SY5Y control cells (Ctrl) and cells oxidized for 2 h with 0.25 and 0.5 mM H_2_O_2_ (OX) in the absence and the presence of 5, 10 and 25 μM arzanol (2 h pre-incubation) and the maximal non-toxic vehicle dose (DMSO 0.25%) (**a**). Scale bar of microscopy images = 100 μm. Intensity of PI red emission fluorescence, expressed as % control, as obtained by image analysis (**b**). All data are presented as mean and standard deviation (SD) of three independent experiments involving duplicated analyses for each sample (n = 6). The statistical significance of differences was assessed by two-way ANOVA followed by the Tukey multiple comparisons test. For each series, *** = *p* < 0.001, ** = *p* < 0.01, and * = *p* < 0.05 versus the respective control cells (Ctrl); °°° = *p* < 0.001 and ° = *p* < 0.05 versus the respective OX samples; ^$$$^ = *p* < 0.001 versus 5 μM arzanol. For each arzanol concentration, ^###^ = *p* < 0.001 and ^#^ = *p* < 0.05 for 0.25 mM versus 0.5 mM H_2_O_2_.

Figure 8a shows the phase contrast and red emission (PI) images measured for differentiated SH-SY5Y control cells and cells oxidized for 2 h, with 0.25 and 0.5 mM H_2_O_2_, in the absence and the presence of arzanol 5, 10 and 25 μM (2 h pre-incubation). The corresponding quantitative data of red fluorescence intensity (expressed as % of control cells) are reported in Figure 8b.

The oxidation of differentiated SH-SY5Y cells for 2 h with 0.25 and 0.5 mM H_2_O_2_ determined a significant (*p* < 0.05) increase in the number of red cells in comparison to control cells (161 and 154% of control for 0.25 and 0.5 mM H_2_O_2_, respectively), as revealed by the PI fluorescence (Figure 8b). Moreover, in H_2_O_2_-oxidized cells an associated altered cell morphology (by phase contrast) was observed, indicated by the presence of rounded cells and cell debris because of cell death.

Significant differences in the arzanol protective effect were observed at the two H_2_O_2_ concentrations.

A significant (*p* < 0.001) protective effect against 0.25 mM H_2_O_2_-induced apoptosis was observed in differentiated neuronal cells pre-incubated for 2 h with 10 and 25 μM arzanol concentrations. The arzanol protection was less evident during cell oxidation with 0.5 mM H_2_O_2_ and a significant (*p* < 0.05) reduction of the red fluorescence was observed only at the highest tested dose of the phloroglucinol (25 μM).

The vehicle DMSO (0.25%) did not exert any protection against the H_2_O_2_-induced apoptosis by PI assay (Figure 8a).

Then, the effect of arzanol against H_2_O_2_-induced apoptosis was assessed by staining differentiated SH-SY5Y cells with NV.

**Figure 9:**
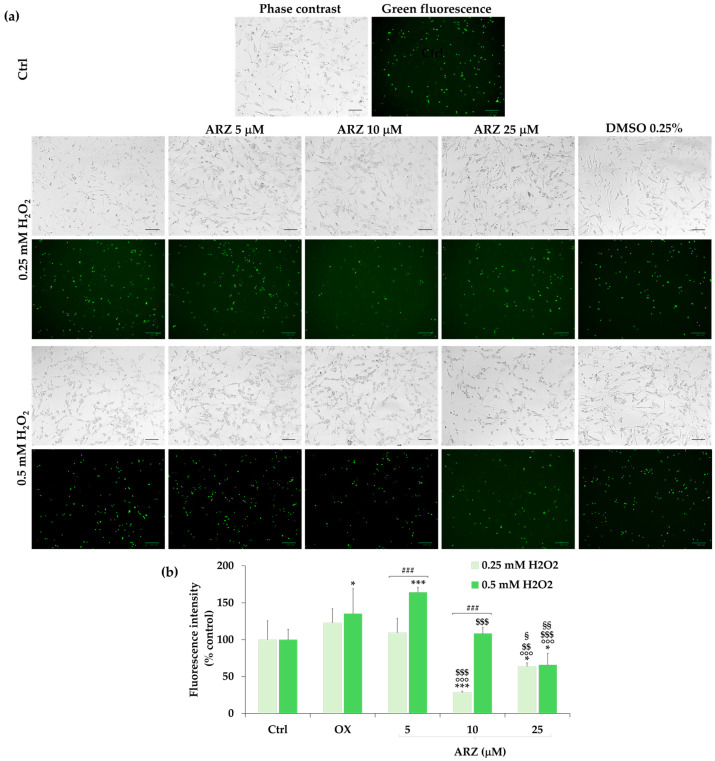
Phase contrast and fluorescence green emission images, by NucView 488 (NV) assay, obtained in differentiated SH-SY5Y control cells (Ctrl) and cells oxidized for 2 h with H_2_O_2_ 0.25 and 0.5 mM (OX) in the absence and the presence of arzanol 5, 10 and 25 μM (2 h pre-incubation) and the maximal non-toxic vehicle dose (DMSO 0.25%) (**a**). Scale bar of microscopy images = 100 μm. The intensity of NV green emission fluorescence, expressed as % control, as obtained by image analysis (**b**). All data are presented as mean and standard deviation (SD) of three independent experiments involving duplicate analyses for each sample (n = 6). The statistical significance of differences was assessed by two-way ANOVA followed by the Tukey multiple comparisons test. For each series, *** = *p* < 0.001 and * = *p* < 0.05 versus the respective control cells (Ctrl); °°° = *p* < 0.001 versus the respective OX samples; ^$$$^ = *p* < 0.001 and ^$$^ = *p* < 0.01 versus 5 μM arzanol; ^§§^ = *p* < 0.01 and ^§^ = *p* < 0.05 versus 10 μM arzanol. For each arzanol concentration, ^###^ = *p* < 0.001 for 0.25 mM versus 0.5 mM H_2_O_2_.

Figure 9a,b show the images of phase contrast/NV green emission and the corresponding green fluorescence intensity data (% of control cells), respectively, measured for differentiated SH-SY5Y control cells, cells oxidized for 2 h with 0.25 and 0.5 mM H_2_O_2_ alone and after 2 h pre-incubation with arzanol (5, 10 and 25 μM).

Significant differences in the arzanol antiapoptotic effect were observed at the two H_2_O_2_ concentrations.

The 2 h oxidation of differentiated SH-SY5Y cells with 0.5 mM H_2_O_2_ determined a significant (*p* < 0.05) increase (135%), in comparison to control cells, in the number of apoptotic cells (green signal) (Figure 9b). A certain pro-apoptotic effect (123% of green fluorescence versus controls) was also observed at 0.25 mM H_2_O_2_.

The 2 h treatment with arzanol 10 and 25 μM induced a significant (*p* < 0.001) marked decrease in the green fluorescence signal versus 0.25 mM H_2_O_2_-oxidized cells, indicating anti-apoptotic properties.

A significant (*p* < 0.001) protective effect of the compound versus differentiated SH-SY5Y cells oxidized with H_2_O_2_ 0.5 mM was observed only at 25 μM.

The vehicle DMSO (0.25%) did not exert any protection against the H_2_O_2_-induced apoptosis by NV assay (Figure 9a).

### 2.7. In Silico Evaluation of Physicochemical and Pharmacokinetic Properties of Arzanol

In silico evaluation of arzanol physicochemical and pharmacokinetic properties was performed using web tools to estimate the potential bioavailability and blood–brain barrier (BBB) permeability for in vivo application in NDs. The arzanol physicochemical properties such as molecular weight (MW), logarithm of the n-octanol/water partition coefficient (XLogP3-AA), number of hydrogen bond donors (HBD) and acceptors (HBA), and topological polar surface area (TPSA), obtained from the PubChem web database [44], are reported in Table S1, while the pharmacokinetic properties calculated with the freely accessible web tools Swiss-ADME [45] and pkCSM-pharmacokinetics [46], using its canonical smiles, are reported in Table S2.

Values of 3.9 (XLogP3-AA) and 3.42 (Consensus Log P_o/w_) for lipophilicity, values of Log S (water solubility) from −4.60 to −6.28, a topological polar surface area (TPSA) of 124 Å², and a total number of hydrogen bonds (THB, donor + acceptor bonds) of 11, were computed for arzanol, indicating mid-polarity properties of the compound [29,44,45].

A high bioavailability was predicted for arzanol by both tools, and a value of 74.92% was estimated for its gastrointestinal absorption (GI) [46]. No BBB permeability (BOILED-Egg model) was predicted for arzanol by Swiss-ADME, while values of −1.204 (log BB) and −2.935 (log PS) were calculated for BBB and CNS permeability, respectively, by pkCSM-pharmacokinetics tool.

## 3. Discussion

Oxidative stress and ROS dysregulation have been implicated in various NDs [1,2,3,4,5]. The high oxygen consumption, weak antioxidant systems, and less cellular regeneration capacity make the central nervous system (CNS) particularly vulnerable to oxidative stress [3,12]. Neuroprotection aims to prevent the loss of neurons, promote the regeneration of neuronal networks, and alleviate brain dysfunction by using agents able to inhibit pathophysiological injurious pathways [19]. Actual research focuses on the individuation of antioxidants able to effectively scavenge free radicals and counteract CNS oxidative stress [3,4,5,13,47]. Antioxidants, such as vitamin C, glutathione, carotenoids, coenzyme Q, vitamin E, and melatonin, have been proposed as preventive and therapeutic molecules in NDs [3,4]. Herbal extracts and natural antioxidant phenols (resveratrol, apigenin, quercetin, curcumin, hesperidin, pectolinarin, and silibinin) with antioxidant properties showed the ability to mitigate oxidative stress-induced damage to neuronal cells and have been proposed as a potentially beneficial strategy in the NDs amelioration/treatment [4,13,15,17,18,19,47,48,49].

Among phenolic compounds, the natural plant-derived heterodimeric phloroglucinyl α-pyrone arzanol is endowed with a wide range of pharmacological properties, including a noticeable antioxidant activity [26,27,29,30]. In the current study, the antioxidant effect of arzanol was evaluated, for the first time, in human differentiated SH-SY5Y cells against the H_2_O_2_-induced oxidative stress. Arzanol showed the ability to preserve neuronal SH-SY5Y cells from H_2_O_2_-induced viability reduction, morphological changes, marked ROS generation, apoptosis, and necrosis.

The SH-SY5Y neuroblastoma cell line is a well-established in vitro model to study NDs and neurotoxicity [3,4,9,37,38,40,41]. The ability of this cell type to differentiate, compared with other models, makes it one of the rare suitable models in neurobiology without having to rely on the primary culture of neuronal cells [14,37,38,40,41,43]. The H_2_O_2_-mediated oxidative stress model has been previously used to evaluate the neuroprotective effects of drugs and natural compounds against ROS-induced neuronal damage and apoptosis both in undifferentiated [9,43,48,49,50,51] and differentiated neuronal SH-SY5Y cells [14,32,43,50,51,52]. In this study, the SH-SY5Y cells were used in two different phenotypes, undifferentiated and differentiated cells, characterized by different biological characteristics [37,41,43].

Arzanol was assessed for cytotoxicity (at 2 and 24 h of incubation) in SH-SY5Y cells by MTT assay. This mitochondrial enzyme-dependent colorimetric test has been previously used to evaluate the cytotoxic and antiproliferative effects of various compounds and extracts in differentiated and undifferentiated SH-SY5Y cells [9,14,32,40,41,50,51,52]. Moreover, we previously used this assay to evaluate the arzanol effect on viability in various cell types (VERO, cancer CaCo-2, HeLa, B16F10, and HaCaT cells) [26,27,29,30]. The pyrone was not significantly toxic in differentiated neuronal SH-SY5Y cells in the range of 2.5–50 μM after 24 h treatment; however, a cytotoxic effect was observed at the highest dose. Interestingly, a significant increase in cell viability, coupled with a certain increased number of fusiform and branched cells, was observed in cells treated for 24 h with arzanol 5–25 μM versus control cells. The increase in cell viability in the range of 5–25 μM was also evident during the short incubation period (2 h). On the contrary, arzanol did not reduce cell viability in undifferentiated SH-SY5Y cells, without causing changes in cell morphology during both periods of incubation. Arzanol exhibited no cytotoxicity in various normal cells (differentiated CaCo-2, HaCaT, and Vero) [29,30], showing the ability to decrease viability in cancer cells (A549, RT-112, HeLa, CaCo-2, and B16F10) [22,23,27].

Our results show a different response of differentiated neuronal SH-SY5Y cells related to the arzanol dose, highlighting a selective potential metabolic stimulation induced by arzanol at 5–25 μM. RA is the most widely used agent for the differentiation of SH-SY5Y cells, and its addition (generally 10 µM) in the culture medium causes rapid differentiation including morphological transformation from neuroblastic- to mature neuron-like cells, as well as decreased cell proliferation [37,53]. A previous study evidenced the role of pro-oxidant effects of RA (production of radicals) in the differentiation of SH-SY5Y cells into an adult neuronal phenotype [53]. The increase in cell viability detected in arzanol-treated cells could probably be ascribable to a potential protective effect against RA-induced oxidative stress, as previously demonstrated for the antioxidant Trolox^®^ [51]. Moreover, a higher cytotoxic effect of arzanol observed in differentiated SH-SY5Y cells than in undifferentiated could be attributed to the increased vulnerability of differentiated cells, similar to mature neurons [37,38].

Then, the cytotoxic effect of supraphysiological [7,8] H_2_O_2_ concentrations was determined in differentiated and undifferentiated SH-SY5Y cells after 2 h incubation. In our experimental conditions, H_2_O_2_ exerted a concentration-dependent cytotoxic effect in differentiated neuronal cells from 0.5 mM (35% viability reduction) to 2 mM, coupled to significant changes in cell morphology (reduction in cellular size and cell number). Values of viability that decreased in the range of 50–60% were previously measured in RA-differentiated SH-SY5Y cells treated, for 24 h, with 0.5 mM of H_2_O_2_ [32,43]. In this study, the peroxide was significantly more cytotoxic in undifferentiated than in differentiated SH-SY5Y cells. A viability reduction of 50% was previously observed after 24 h treatment with H_2_O_2_ 0.375 mM in undifferentiated SH-SY5Y cells [43]. A significant dose-dependent viability reduction was reported in human neuroblastoma SH-SY5Y cells exposed for 12 h to H_2_O_2_ in the 0.2–1 mM range [48].

Our results show that the pre-incubation for 24 h with arzanol protected differentiated SH-SY5Y cells from H_2_O_2_-induced injury (by MTT assay). Furthermore, the cell morphology was also restored to normal by arzanol treatment. A certain protection was also observed at 2 h of incubation with the compound. A previous study evidenced that the pre-treatment (24 h) with arzanol preserved HaCaT cells from cytotoxicity induced by incubation for 2 h with H_2_O_2_ [30]. It was found that H_2_O_2_ lowered differentiated SH-SY5Y cell viability by increasing ROS production and inducing abnormal changes in cell morphology, mitochondrial dysfunction, and ROS mitochondria-mediated apoptosis [14,32,43,50,51,52]. Similar effects were also triggered by the peroxide in neuroblastoma SH-SY5Y cells [9,43,48,49,50,51]. In our experimental conditions, the lower protective effect of arzanol, against H_2_O_2_-induced cytotoxicity, in undifferentiated SH-SY5Y cells than in differentiated SH-SY5Y could probably be attributable to the higher cytotoxicity of the oxidant in undifferentiated cells.

The toxic action of high H_2_O_2_ concentrations is mediated by a marked ROS generation [47]. In this study, the treatment of differentiated SH-SY5Y cells with H_2_O_2_ 0.5 mM significantly induced a cellular ROS increase during 2 h of exposure, compared to the basal level of control cells, according to previous studies [14,32,43]. Pre-incubation for 2 h with arzanol significantly reduced ROS production in comparison to H_2_O_2_-oxidized neuronal SH-SY5Y cells at all tested antioxidant concentrations, confirming the antioxidant properties of this phloroglucinol. The ability of arzanol (24 h of pre-treatment) to protect against ROS generation induced by 1 h of exposure to H_2_O_2_ (0.5–5 mM) was previously evidenced in HaCaT cells [30]. The comparative evaluation of the arzanol effect against H_2_O_2_-induced ROS production in undifferentiated cells evidenced a lower protective effect than in differentiated cells, inversely correlated to the compound amount. Interestingly, the treatment with the only compound did not significantly affect the ROS basal level, in both differentiated and undifferentiated SH-SY5Y cells.

Studies have highlighted the role of oxidative stress and associated mitochondrial dysfunction in the pathophysiologic processes involved in NDs [1,3,4]. The oxidative stress/ROS generation impacts mitochondria and can cause damage through apoptosis [19]. High H_2_O_2_ concentrations have been demonstrated to promote cell apoptosis in the brain, leading to NDs [32,47]. Therefore, the protective effect of arzanol against the apoptosis induced by H_2_O_2_ in differentiated SH-SY5Y cells was evaluated by NV assay, able to detect the activity of caspase-3/7 inside cells [40], and PI assay, which evidences necrotic and late apoptotic cells [43]. In our experimental conditions, the 2 h incubation of differentiated SH-SY5Y cells with H_2_O_2_ (0.25 and 0.5 mM) caused an increase in apoptotic/necrotic rate in comparison to control cells (NV and PI assays). The presence of apoptotic rounded cells and cell debris from cell death was confirmed in H_2_O_2_-oxidized cells by phase contrast microscopy. An increase in apoptotic cells was previously reported in differentiated SH-SY5Y cells treated for 2 h with 100 μM H_2_O_2_ by the use of acridine orange/ethidium bromide dyes [14] and for 4 h with 500 μM H_2_O_2_ by Annexin V-propidium iodide [50]. The treatment of differentiated SH-SY5Y cells with arzanol exerted a strong significant protective effect against H_2_O_2_-induced apoptosis/necrosis. The anti-apoptotic effect of arzanol (24 h of pre-treatment, 50 μM) was previously demonstrated in HaCaT cells exposed for 1 h to H_2_O_2_ (0.5–5 mM) [30]. Interestingly, a significant decrease in apoptotic/necrotic cells, compared to the basal level of control cells, was observed in differentiated SH-SY5Y cells incubated for 2 h with only arzanol, evidencing its protection against the physiological apoptotic process present in differentiated SH-SY5Y cells.

Our data showed that arzanol was able to protect differentiated SH-SY5Y cells (undifferentiated cells were partially protected) against oxidative damage induced by high (supraphysiological) concentrations of H_2_O_2_ (250 and 500 μM) in the range 5–25 μM (values of arzanol:H_2_O_2_ ratios from 1:10 to 1:100), and its activity was comparable to that previously observed for other natural and synthetic antioxidants in this neuronal oxidative stress model [9,14,32,43,47,48,50,54]. Arzanol showed the ability to preserve neuronal SH-SY5Y cells from H_2_O_2_-induced viability reduction, morphological changes, marked ROS generation, and ROS-mediated apoptosis and necrosis.

High (supraphysiological) concentrations of H_2_O_2_ have been widely used in neuronal experimental cell models to induce oxidative stress-related damages [47,48,49]. Due to its small size and lack of charge, H_2_O_2_ crosses cell membranes easily and diffuses inside/outside the cells [33,47]. Its toxic action mode is mediated by the activation of N-methyl-D-aspartate receptors that leads to massive Ca^2+^ influx, oxidative stress induced by marked ROS generation, and cell apoptosis [47]. The H_2_O_2_ entry in neuronal cells generates highly reactive hydroxyl radicals (HO^•^) by Fenton’s reaction, spreading the HO^•^-induced damage [10,49]. The peroxide and the Fenton’s reaction byproducts can directly attack and destroy cellular proteins, DNA, lipids, and mitochondria [47]. The interaction between HO^•^ radicals and biological structures results in their destruction/oxidation with the generation of oxidative products, massive ROS generation, viability reduction, cell death, morphological changes, mitochondrial damage, and apoptosis [9,14,49].

In our experimental conditions, neuronal SH-SY5Y cells were pre-incubated with non-cytotoxic doses of arzanol, and the compound was then removed before H_2_O_2_ cell exposure. Previous studies evidenced the noteworthy antioxidant properties of arzanol in chemical systems of lipid peroxidation (cholesterol, linoleic acid, liposomal membranes and lipoproteins) and cultured cell/animal models of oxidative stress [26,27,29,30]. The arzanol antioxidant properties have been correlated to its efficacy in scavenging lipid peroxyl (ROO^•^) and HO^•^ radicals, acting as hydrogen atom donor from hydroxyl groups (chain-breaking antioxidant) [26,27,29,30]. A metal ion chelation ability has also been proposed for arzanol protection against Cu^2+^-induced oxidative damage of liposome vesicles and human low-density lipoproteins [27,29]. Previous studies, conducted in monolayers of colon differentiated CaCo-2 cells, an intestinal cell model, evidenced the bioavailability of arzanol and its capacity to cross biological membranes mainly by a passive diffusion pathway [29]. Moreover, in silico determination by SwissADME and pkCSM-pharmacokinetics [45,46] confirmed the high bioavailability of arzanol, due to its mid-polarity properties [29].

Neuronal SH-SY5Y cells pre-incubated with arzanol and then treated with high H_2_O_2_ concentrations exhibited a marked reduction in H_2_O_2_-induced ROS increase. Therefore, the arzanol protective effect could be partially mediated by a direct scavenging action on HO^•^ and LOO^•^ radicals inside cells or in the phospholipid bilayers of cell membranes, in which the compound could insert for its chemical and physical properties (lipophilicity, TPSA, and THB), as reported for other phenolic compounds [55].

High H_2_O_2_ concentrations have been demonstrated to promote cell apoptosis by massive ROS generation that impacts mitochondria [19,32,47]. In our experimental conditions, the treatment of SH-SY5Y cells with H_2_O_2_ induced an increased apoptotic process (NV and PI assays) [40], and an increased number of apoptotic and necrotic cells were detected. Arzanol significantly protected neuronal SH-SY5Y against H_2_O_2_-induced apoptosis probably through the reduction of the H_2_O_2_-induced massive ROS generation, as previously observed in HaCaT cells [30].

It has been reported that the activation of the transcription factor NF-kB is involved in the pathogenesis of several NDs and increased in dopaminergic neurons of PD patients [56]. A previous study demonstrated the upregulation of TNF-α and NF-κB protein expression in H_2_O_2_-treated SH-SY5Y cells, indicating that H_2_O_2_ could activate inflammatory responses [57]. Moreover, some authors reported the pro-apoptotic role of NF-kB activation [56]. Arzanol is a well-established anti-inflammatory compound and has been reported to inhibit inflammatory transcription factor NF*κ*B activation [21,28]. Therefore, the capacity of arzanol to reduce H_2_O_2_-mediated apoptosis by suppressing NF-κB activation could not be excluded.

**Scheme 1:**
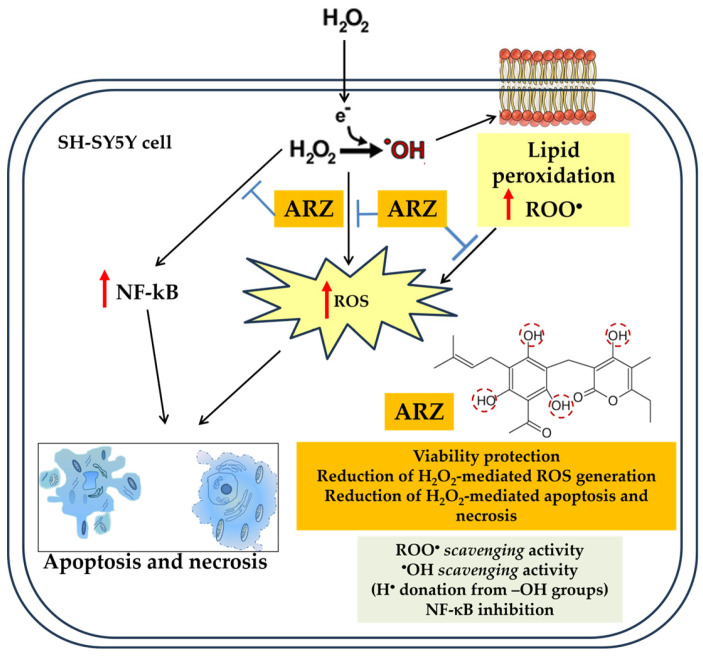
Possible mechanism of arzanol action against H_2_O_2_-mediated oxidative stress in neuronal SH-SY5Y cells.

A summary of the possible mechanism of action of arzanol against H_2_O_2_-mediated oxidative stress in neuronal SH-SY5Y cells is reported in Scheme 1.

The permeability study of bioactive molecules across the BBB is considered an essential approach to screening for neuroprotective compounds capable of reaching CNS, and molecular parameters, such as lipophilicity, TPSA, and MW, have been demonstrated to affect diffusion across BBB [58]. Molecules with higher log P values have been proposed to penetrate better through the BBB due to the lipophilic nature of this physiological barrier [58]. Cell culture and in silico studies evidenced the bioavailability of arzanol and its ability to cross biological membranes by passive diffusion [29,44,45]; however, no BBB permeability (by passive diffusion) was predicted for arzanol by the Swiss-ADME (BOILED-Egg model) [45]. In the pkCSM-pharmacokinetics model, molecules with a log BB < −1 are considered poorly distributed to the brain, while compounds with values > 0.3 readily cross the BBB [59]. Moreover, compounds with a log PS > 2 are considered to penetrate the CNS, while those with log PS < −3 are considered unable to penetrate the CNS. Values of −1.204 (log BB) and −2.935 (log PS) were calculated for BBB and CNS permeability, respectively, by pkCSM-pharmacokinetics for arzanol, indicating a low computed BBB and CNS permeability. Arzanol was predicted to passively not cross BBB; however, a carrier- or receptor-mediated transport through the BBB cannot be excluded.

## 4. Materials and Methods

### 4.1. Materials

Chemicals for cell culture, including penicillin, streptomycin, fetal bovine serum (FBS), Dulbecco’s Modified Eagle’s Medium (DMEM), and Trypsin 0.25%-EDTA were acquired from EuroClone (Pero, MI, Italy). Retinoic acid (RA), hydrogen peroxide (H_2_O_2_) solution 30% (*w*/*w*), 2′,7′-dichlorodihydrofluorescein diacetate (H_2_DCFDA), 3-(4,5-dimethylthiazol-2-yl)-2,5-diphenyltetrazolium bromide (MTT), and dimethyl sulfoxide (DMSO) were purchased from Merck Life Science (Milan, Italy). Propidium iodide (PI) was acquired from Thermo Fisher Scientific (Waltham, MA, USA), and NucView^®^ 488 (NV) was purchased from Biotium (Fremont, CA, USA).

### 4.2. Arzanol Extraction and Isolation

Arzanol (Figure 1) was extracted and isolated (final purity was 98%) in conformity with the literature [21] from the dried aerial parts (leaves and flowerheads) of *H. italicum* ssp. *microphyllum*, collected in 2021, Sardinia, Italy. A plant batch codified HM-CA2021 is being kept in the phytochemistry laboratory of Novara, Italy. Arzanol was characterized by ^1^H NMR and ^13^C NMR spectroscopic methods, and the structure was confirmed by comparing the data with related scientific literature [21].

### 4.3. SH-SY5Y Cell Culture

Human neuroblastoma cell line SH-SY5Y was supplied by the Cell Bank Interlab Cell Line Collection, IRCCS San Martino Policlinico Hospital, Genova, Italy (code# HTL95013). Neuroblastoma cells, in DMEM high-glucose medium enriched with 10% *v*/*v* heat-inactivated FBS, 100 units/mL of streptomycin/penicillin, were grown in a humidified atmosphere of 5% CO_2_ and 95% humidity at 37 °C. The cells were propagated by changing the medium every two days and were split weekly using Trypsin 0.25%-EDTA when confluent.

### 4.4. Neuronal Differentiation Induction

For the differentiation process, neuroblastoma SH-SY5Y cells were plated (density of 10^5^/mL) and cultured in high-glucose DMEM medium enriched with 2.5% *v*/*v* heat-inactivated FBS, 100 units/mL of streptomycin/penicillin, and 10 μM of RA to induce dopaminergic differentiation (differentiation medium). The medium was changed every 48 h for 7 days before experimental studies. The evaluation of neuronal morphology was assessed by microscopic observation using a ZOE™ Fluorescent Cell Imager (Bio-Rad Laboratories, Inc., Hercules, CA, USA).

### 4.5. Immunofluorescence

The immunofluorescence demonstration of tyrosine hydroxylase enzyme was performed on methanol-fixed undifferentiated and differentiated cells to confirm the neuronal dopaminergic differentiation of SH-SY5Y neuroblastoma cells. After rehydration in phosphate-buffered saline (PBS) with 0.2% Triton-X-100, the cells were treated with 20% normal goat serum in PBS (30 min). Rabbit polyclonal antibody to human tyrosine hydroxylase (TH) (AB152, Merck Millipore, Burlington, MA, USA) was used as a primary antiserum. Alexa Fluor 594-conjugated polyclonal goat anti-rabbit antibodies (A-11012, Invitrogen, Waltham, MA, USA) were used as secondary antisera. Cells were rinsed thoroughly in PBS-Triton between each step, counterstained with Hoechst-33342 for nuclei detection, and finally mounted in PBS-glycerol. In negative control cells, the antisera specificity was tested by replacing the primary antibody with normal serum.

### 4.6. Cell Viability (MTT Assay)

#### 4.6.1. Effect of H_2_O_2_ and Arzanol on Cell Viability

The cytotoxic effect of arzanol, DMSO (vehicle molecule), and H_2_O_2_ on the differentiated SH-SY5Y cell viability was evaluated by MTT assay, as previously reported [30]. SH-SY5Y neuroblastoma cells, plated (at 10^5^ cells/mL density) in a 96-well plate, were induced to differentiate into dopaminergic neuronal cells. Then, the differentiated cells were incubated with arzanol (2.5–100 μM, from a 10 mM solution in DMSO) in a complete differentiation medium for 2 and 24 h. After medium removal, the MTT assay was performed as previously reported [30]. The absorbance of the formazan crystals was measured at 570 nm with an Infinite 200 microplate reader (Tecan Sales Austria GmbH, Grödig, Salzburg, Austria). The viability of arzanol-treated cells was compared to that of the control (untreated) cells. The absorbance of control cells was evaluated as 100% alive and viability data were reported as the percentage of control cells. The effect on cell viability of DMSO (from 0.025 to 1%), the molecule vehicle of the phenolic compound, was also determined.

The H_2_O_2_ impact on differentiated cells was assessed in a separate series of experiments. After differentiation, cells were incubated for 2 h with various H_2_O_2_ concentrations (ranging from 0.0125 to 2 mM) in a complete differentiation medium and then subjected to the MTT assay as previously described.

Moreover, the cytotoxicity of arzanol and H_2_O_2_ was evaluated in undifferentiated SH-SY5Y cells for comparison. Neuroblastoma SH-SY5Y cells were plated at a density of 10^5^ cells/mL in a 96-well plate and cultured for 48 h in a complete DMEM medium. Then, the undifferentiated cells were separately treated with arzanol and H_2_O_2_ and subjected to MTT assay, according to the same experimental design of differentiated cells.

The cell morphology was evaluated by microscopic observation using a ZOE™ Fluorescent Cell Imager (Bio-Rad Laboratories, Inc., Hercules, CA, USA).

#### 4.6.2. Protective Effect of Arzanol against H_2_O_2_-Induced Neuronal Cytotoxicity

Neuroblastoma cells were plated at a density of 10^5^ cells/mL in a 96-well plate and induced to differentiate into dopaminergic neuronal cells. After the differentiation process, the cells were subjected, in fresh medium, to four experimental sets: control cells; cells pre-incubated for 2 and 24 h with arzanol (5, 10, and 25 μM); cells incubated with 0.25 and 0.5 mM H_2_O_2_ in DMEM (2 h incubation); cells pre-treated with arzanol and then exposed to H_2_O_2_ oxidation. Then, all cell groups were subjected to MTT assay [30].

Viability data were expressed as a percentage of respective control cells. DMSO effect at the maximal amount (0.25%) was also evaluated. The morphology of the differentiated neurons was microscopically observed at every stage.

The arzanol protection against H_2_O_2_-viability reduction was also monitored by MTT assay in undifferentiated SH-SY5Y neuroblastoma cells. SH-SY5Y cells were plated at a density of 10^5^ cells/mL in a 96-well plate and cultured for 48 h in a complete medium. Then, cells were subjected to four experimental sets, as reported for differentiated cells.

### 4.7. H_2_O_2_-Induced ROS Generation

The H_2_DCFDA assay was used to determine ROS generation in neuronal differentiated cells exposed to H_2_O_2_ with or without arzanol treatment [30]. The H_2_DCFDA assay is based on the structure of DCF, which interacts with reactive species and leads to fluorescence [60]. H_2_DCFDA can penetrate the cells, and cytoplasmic esterases cleave its acetyl groups, releasing DCF-H_2_ that reacts with ROS and fluoresces [42]. SH-SY5Y neuroblastoma cells, seeded in 96-well plates at 10^5^ cells/mL density in 100 μL/well, were differentiated in neuronal cells. Then, the cells were incubated for 2 h in a fresh medium in the absence (control untreated cells) and in the presence of arzanol (5, 10, and 25 μM). The cells, after washing, were incubated (30 min) in the presence of 10 μM H_2_DCFDA in PBS at 37 °C. After removing the H_2_DCFDA solution, neuronal cells were exposed to H_2_O_2_ 0.5 mM in PBS, while control cells were incubated with PBS alone. DMSO effect at the maximal amount (0.25%) was also evaluated against 0.5 mM H_2_O_2_-mediated ROS generation. An Infinite 200 Tecan microplate reader was used to monitor ROS generation (every 5 min at 37 °C) during 2 h of exposure to H_2_O_2_. The fluorescence data, obtained at 490 nm (wavelength of excitation) and 520 nm (wavelength of emission) were processed using Tecan I-control 1.5V software, and normalized to control cells.

The same experimental H_2_DCFDA protocol was applied to undifferentiated neuroblastoma SH-SY5Y cells for comparison.

### 4.8. Propidium Iodide and NucView Apoptosis Assays

The arzanol protection against cell apoptosis and death induced by H_2_O_2_ oxidation was assessed in differentiated SH-SY5Y neuronal cells by PI and NV assays, as previously reported [30,40]. PI is a fluorescent dye that cannot enter living cells and intercalates between DNA base pairs of the necrotic and late apoptotic cells that lost the cell membrane integrity [43]. NV is a combination of a fluorogenic DNA dye and a DEVD substrate moiety, a complex initially non-fluorescent and non-functional. Once it reaches the cytoplasm, it can be cleaved by caspase-3/7 releasing a high-affinity DNA dye that dyes DNA in the nucleus with green fluorescence and profiles apoptotic cells [30,40].

SH-SY5Y neuroblastoma cells were seeded in 96-well plates at 10^5^ cells/mL density in 100 μL/well of complete culture medium. After the differentiation process, the cells were subjected, in fresh medium, to different experimental sets: control cells, cells treated for 2 h with arzanol (5, 10, and 25 μM), cells 2 h oxidized with 0.25 and 0.5 mM H_2_O_2_, and cells pre-treated with arzanol or the corresponding amount of the vehicle (0.25% of DMSO) and then exposed to H_2_O_2_. After, the neuronal cells were incubated with PI (final concentration 1 μg/mL) and, in a separate set of samples, with NV, according to the guidelines provided by the manufacturer. After dark incubation with PI (1 h) and NV (2 h), microscopic observations were made using a ZOE™ Fluorescent Cell Imager, adjusting offset values and instrument gain using control cells. Evaluation of PI and NV images was performed by ImageJ software (version 1.53e). Background fluorescence was subtracted from images, and histogram values of fluorescence intensity were expressed as % of control cell fluorescence. Five images were processed for each sample.

### 4.9. In Silico Evaluation of the Physicochemical and Pharmacokinetic Properties of Arzanol

Physicochemical properties (MW, XLogP3-AA, TPSA, HBD, HBA and TPSA) of arzanol were obtained from PubChem, a free web database [44]. The molecular structure of arzanol was introduced in simplified molecular-input line entry specification (SMILES) nomenclature into the freely accessible web tools SwissADME and pkCSM-pharmacokinetics. The SwissADME web tool [45], which provides a global evaluation of the pharmacokinetics profile of small molecules [59], was used to estimate arzanol lipophilicity (Consensus Log P_o/w_), water solubility (Log S), GI absorption (according to the white of BOILED-Egg model), and BBB permeation (according to the yolk of BOILED-Egg model). The pkCSM-pharmacokinetics web tool [46], a novel method for predicting and optimizing small-molecule pharmacokinetics properties [61], was employed to calculate arzanol intestinal absorption (%), the logarithm of the ratio of arzanol concentration in the brain and the blood (log BB), and the logarithm of blood–brain permeability-surface area product (log PS).

### 4.10. Statistical Analyses

Results were expressed as mean and standard deviation (SD) of three independent experiments involving multiple analyses for each sample. GraphPad Prism version 10.0.0 for Windows (GraphPad Software, Boston, MA, USA) was used to estimate the statistical differences between different groups of data. Multiple comparisons of the group means were evaluated by two-way analysis of variance (two-way ANOVA) followed by the Tukey multiple comparisons test. The minimal level of significance was *p* < 0.05.

## 5. Conclusions

In this study, for the first time, the natural plant-derived arzanol showed a noteworthy antioxidant effect against H_2_O_2_-induced oxidative damage in differentiated SH-SY5Y cells, a neuronal cell model widely used to study NDs. Arzanol showed the ability to preserve neuronal SH-SY5Y cells from H_2_O_2_-induced viability reduction, morphological changes, ROS generation, and apoptosis/necrosis. Our results furnish useful indications about the potential use of arzanol in the development of future therapeutic strategies in the treatment of ND-related oxidative stress conditions.

The limitations of this study were the use of only one neuronal cell line, the lack of kinetic analysis of hydrogen peroxide consumption, and the lack of evaluation of antioxidant enzymes and proteins associated with both apoptosis and anti-apoptosis. Hence, additional research is required to gain a better understanding of the arzanol protective mechanisms. These studies should encompass other neuronal cell lines and mouse brain models using different methods to assess the viability and the impact of H_2_O_2_-induced oxidative stress. Finally, in vivo translation of these results will require the assessment of the penetration abilities of arzanol or its semi-synthetic derivatives (able to overcome its pharmacokinetic limits) through the BBB and a clear definition of the transport mechanism.

## Data Availability

The data that support the findings of this study are available from the corresponding author upon reasonable request.

## Supplementary Materials

The following supporting information can be downloaded at: https://www.mdpi.com/article/10.3390/ijms25137386/s1.
