# Supplementary material for: Protective Effect of Arzanol against H2O2-Induced Oxidative Stress Damage in Differentiated and Undifferentiated SH-SY5Y Cells
Source: Int J Mol Sci. 2024 Jul 5;25(13):7386. doi: 10.3390/ijms25137386 (PMC11242736; doi:10.3390/ijms25137386)
Supplement: Supplementary file 1 [file ijms-25-07386-s001.zip › ijms-3065497-supplementary.pdf]

Supplementary Materials

Table of Contents

Figure S1: Phase contrast images of undifferentiated SH-SY5Y control cells after 24 h incubation with arzanol.

Figure S2: ROS-induced fluorescence measured for 2 h in differentiated and undifferentiated SH-SY5Y cells after 2 h-pre-incubation with arzanol.

Table S1: Physicochemical properties of arzanol computed with the PubChem database.

Table S2: Pharmacokinetic properties of arzanol calculated with the web tools SwissADME and pkCSM-pharmacokinetics.

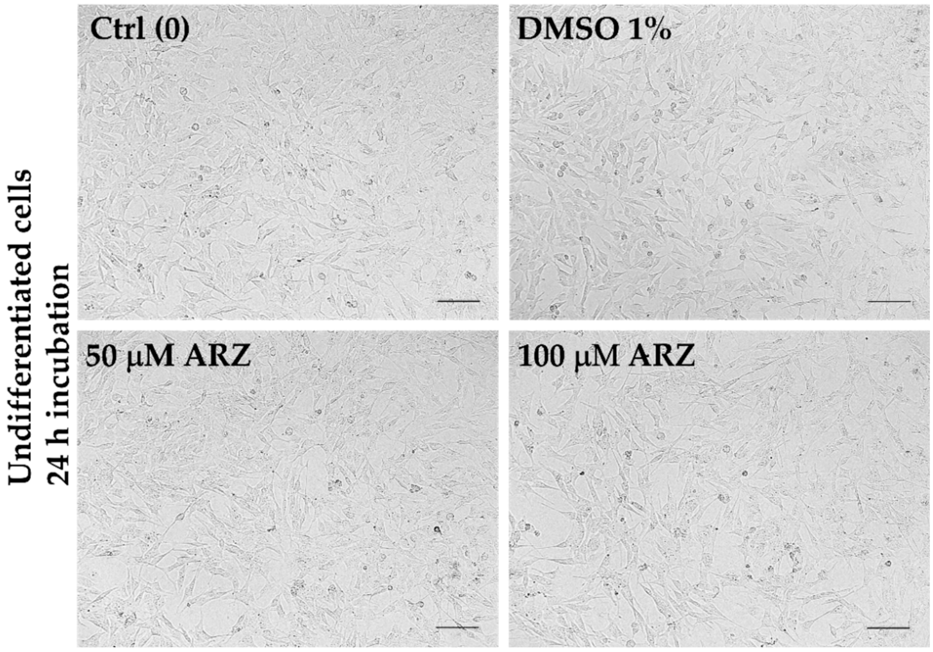

Figure S1. The panel shows representative phase contrast images of undifferentiated SH-SY5Y control cells (Ctrl), and cells treated for 24 h with vehicle (DMSO 1%) and arzanol (50 and 100 μM). Bar = 100 μm.

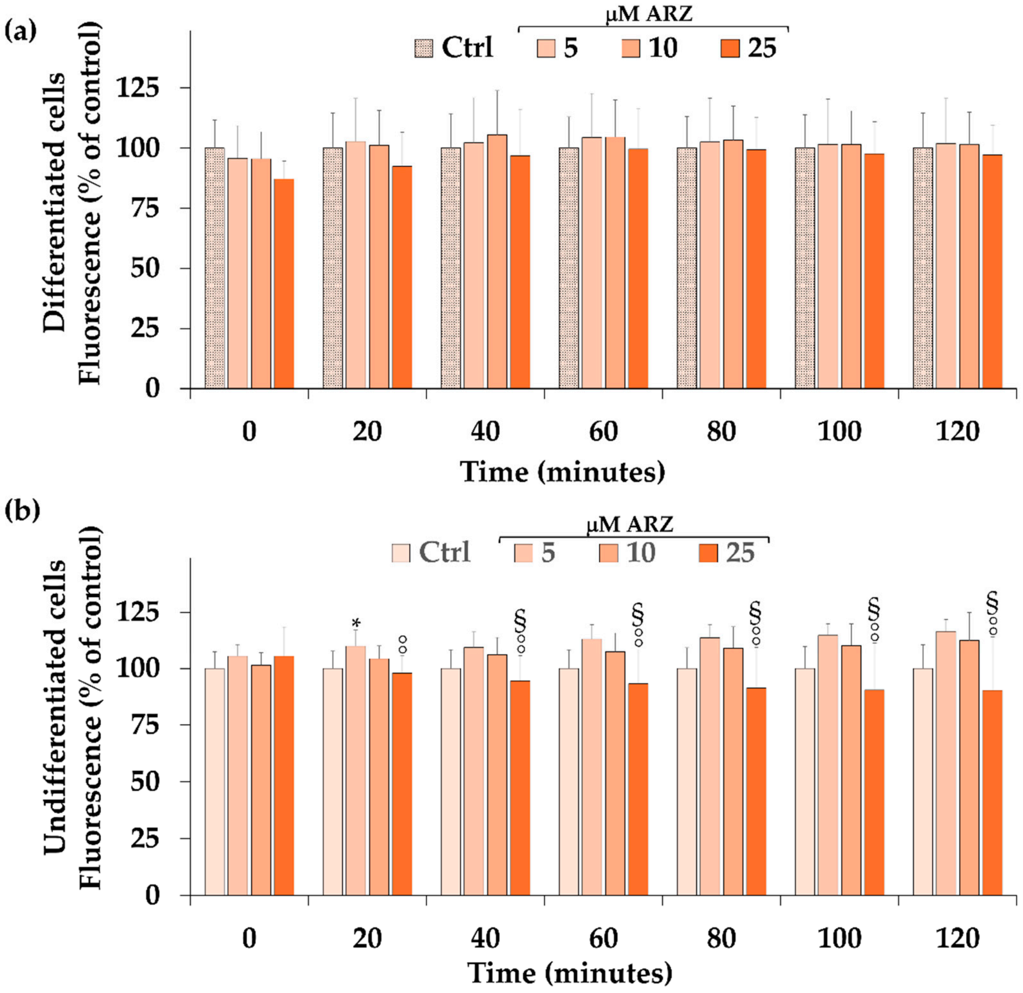

Figure S2. ROS-induced fluorescence, expressed as % of the control (0), measured for 2 h, at different time points, in control cells (0) and cells after 2 h-pre-incubation with arzanol (5, 10, and 25 µM) in differentiated (**a**) and undifferentiated (**b**) SH-SY5Y cells. All data are presented as mean and standard deviation (SD) of three independent experiments involving five replicates for each sample (n = 15). The statistical significance of differences was assessed by Two-way ANOVA followed by the Tukey Multiple Comparisons Test. At each time point: * = $p < 0.05$ versus the respective control; °° = $p < 0.01$ versus 5 µM arzanol; § = $p < 0.05$ versus 10 µM arzanol.

Table S1. Physicochemical properties of arzanol computed from the chemical structure, obtained from the PubChem database [44].

Computed property	Reference (computed by)	Value
Molecular Weight	PubChem 2.1	402.4 g/mol
XLogP3-AA	XLogP3 3.0	3.9
Hydrogen Bond Donor Count	Cactvs 3.4.6.11	4
Hydrogen Bond Acceptor Count	Cactvs 3.4.6.11	7
Topological Polar Surface Area	Cactvs 3.4.6.11	124Å^2

Table S2. Pharmacokinetic properties of arzanol calculated with the web tools SwissADME [45] and pkCSM-pharmacokinetics [46] using the canonical smiles of arzanol [1].

Property	Model Name	Value
	Swiss-ADME	
Lipophilicity	Consensus Log Po/w	3.42
Water Solubility	Log S (ESOL)	-4.70
Water Solubility	Log S (Ali)	-6.28
Water Solubility	Log S (SILICOS-IT)	-5.26
Gastrointestinal (GI) absorption	BOILED-Egg (white)	High
Blood brain barrier (BBB) permeant	BOILED-Egg (yolk)	No
	pkCSM-pharmacokinetics	
Absorption	Intestinal absorption (human)	74.92%
Distribution	BBB permeability	-1.204 (log BB)
Distribution	CNS permeability	-2.935 (log PS)

[1] CCC1=C(C(=C(C(=O)O1)CC2=C(C(=C(C(=C2O)CC=C(C)C)O)C(=O)C)O)O)C [44].
